# Differential diagnosis of benign and malignant ovarian tumors with combined tumor and systemic inflammation-related markers

**DOI:** 10.7150/jca.112768

**Published:** 2025-07-11

**Authors:** Dan Song, Tingting Liu, Chao Han, Chu Zhang, Weimin Kong

**Affiliations:** Department of Gynecology, Beijing Obstetrics and Gynecology Hospital, Capital Medical University. Beijing Maternal and Child Health Care Hospital, Beijing 100026, China.

**Keywords:** epithelial ovarian cancer, diagnosis, inflammatory indicators, tumor markers.

## Abstract

**Background:** To explore the diagnostic significance of pre-surgery peripheral blood tumor markers cancer antigen 125 (CA125) and human epitope protein 4 (HE4), in conjunction with neutrophil-to-lymphocyte ratio (NLR), monocyte-to-lymphocyte ratio (MLR), platelet count-to-lymphocyte ratio (PLR), and systemic immunoinflammatory index (SII), in the differential diagnosis of epithelial ovarian cancer (EOC) and benign ovarian tumors. Determine the best combination of diagnostic indicators for early diagnosis of EOC.

**Methods:** We retrospectively analyzed clinical data from 189 patients with EOC and 202 patients with benign ovarian tumors, comparing levels of CA125, HE4, and inflammatory markers, and evaluated the efficacy of these markers in diagnosing EOC alone or in combination by calculating sensitivity, specificity, and receiver operating characteristic curve (ROC).

**Results:** Serum concentrations of CA125, HE4, NLR, PLR, MLR, and SII were significantly higher in the EOC group than in the benign ovarian tumor group (P < 0.001). In 189 cases of EOC, CA125 and HE4 were significantly higher in advanced stages than in early stages (P = 0.000, P = 0.012). NLR, PLR, MLR, and SII showed no significant difference between early and advanced stages (P>0.05), and this was also the case in 141 patients with high-grade serous ovarian cancer. CA125, HE4, NLR, PLR, MLR, and SII showed no significant differences across age groups, menopausal states, or pathological types (P > 0.05 for all). For diagnosing EOC, both the CA125+HE4+NLR+PLR+MLR+SII and CA125+HE4+PLR+MLR+SII models achieved the highest AUC values (AUC = 0.951 for both). No statistically significant difference was observed between these two models in AUC comparison (P=0.9305). NLR alone showed the lowest AUC at 0.696. The CA125+HE4+PLR+MLR+SII model demonstrated the highest sensitivity (84.66%), while CA125+HE4 showed the highest specificity (95.54%).

**Conclusion:** Preoperative peripheral blood tumor markers combined with inflammatory markers can improve the diagnostic efficiency of EOC. Among these combinations, CA125+HE4+PLR+MLR+SII demonstrated optimal diagnostic performance with the highest efficacy and sensitivity, providing a clinical basis for enhanced EOC diagnosis.

## Introduction

Ovarian cancer (OC) stands as a prevalent malignancy in the female reproductive system, with its death rate being the highest among gynecological tumors globally 1. In the realm of OC, epithelial ovarian cancer (EOC) ranks as the predominant type, representing over 90% of cases. OC is insidious, exhibiting no common early-stage symptoms. OC typically progresses insidiously with no specific early-stage symptoms. Consequently, the majority of OC cases are clinically identified at advanced stages, which correlates with high mortality rates and poor prognosis. In EOC, patients diagnosed at early stages (FIGO I-II) demonstrate a 5-year survival rate exceeding 90%, whereas over 70% present with advanced-stage disease (FIGO III-IV) at initial diagnosis. Patients with advanced EOC have a poor prognosis, with a 5-year survival rate of only 31% 2. Early detection is strongly associated with improved patient outcomes. Although contemporary ovarian cancer management has achieved significant therapeutic advances, reliable early detection techniques are still absent. Therefore, identifying novel clinical biomarkers is critical to improving early diagnosis rates and ultimately enhancing survival outcomes.

Currently, there is no established standardized approach for the early detection of OC. Although cancer antigen 125 (CA125) and human epididymis protein 4 (HE4) remain the most clinically valuable tumor biomarkers for EOC diagnosis, their diagnostic accuracy requires further improvement. OC features a unique tumor microenvironment, consisting of stromal cells, the extracellular matrix, and exosomes. Cancer-associated inflammation critically modulates the tumor microenvironment by orchestrating angiogenesis, regulating tumor cell proliferation/differentiation, and subverting immune surveillance mechanisms that collectively drive tumor initiation, progression, and metastatic dissemination 3. Previous studies have shown that inflammation can affect the pathogenesis and progression of cancer 4, 5. Systemic inflammatory biomarkers, such as monocyte-to-lymphocyte ratio (MLR), neutrophil-to-lymphocyte ratio (NLR), and platelet-to-lymphocyte ratio (PLR), are associated with the diagnosis and outcomes of different tumors 6-9. The systemic immune inflammation index (SII), which integrates platelet, neutrophil, and lymphocyte counts, has emerged as a robust prognostic biomarker across multiple malignancies, including OC 10, 11. Nonetheless, the effectiveness of inflammatory indicators in diagnosing OC requires further external validation.

The potential of inflammation-based scores to differentiate benign from malignant tumors and predict clinical outcomes has attracted growing attention in the international research community. Inflammation-based hematologic indices like NLR, MLR, PLR, SII, and several others, derived from routine complete blood counts, have gained prominence in cancer research due to their standardized measurement, cost-effectiveness, and widespread clinical availability. While many studies utilize CA125 and HE4 either alone or in combination with inflammatory indices, comparative analyses evaluating the diagnostic performance of these indices remain limited. Extensive clinical research is still required to determine the optimal threshold values for each inflammation score. Therefore, this research aimed to explore the diagnostic significance of the CA125 and HE4 combined preoperative systemic inflammation score in the differential diagnosis of EOC and benign ovarian tumors.

## Materials and Methods

### Study population

We conducted a retrospective analysis of 189 patients with EOC and 202 patients with benign ovarian tumors, diagnosed by postoperative pathology at Beijing Obstetrics and Gynecology Hospital, Capital Medical University, between January 2020 and December 2023. Inclusion criteria were (1) EOC patients were classified according to FIGO 2009 criteria (stages I-IV); (2) first-time comprehensive staging/cytoreductive surgery for EOC, oophorectomy/ovarian cystectomy for benign tumors; (3) no prior adjuvant therapy or antibiotic use within 2 weeks before surgery; (4) complete clinical records, preoperative tumor markers within 2 weeks, and standard blood tests within 1 week. Exclusion criteria were (1) experiencing other infectious diseases, active infections, or inflammatory diseases within a month; (2) hematological diseases; (3) immune system disorders; (4) severe hepatic/renal dysfunction; (5) other malignancies. This retrospective study was approved by the Medical Ethics Committee of Beijing Obstetrics and Gynecology Hospital, Capital Medical University.

### Systemic inflammation-related markers

Chronic inflammation and immunosuppression are key drivers of tumorigenesis. The quantitative assessment of the imbalance between pro-tumor inflammation and anti-tumor immunity has demonstrated prognostic value in various solid and hematologic malignancies. Scores based on absolute values of different types of leukocytes include the NLR, MLR, PLR, and SII. The characteristics and clinical significance of these inflammatory markers are summarized below (Table 1).

### Data collection

Clinical data were collected from all patients, including (1) demographic characteristics (age, BMI, menopausal status); (2) preoperative laboratory results (complete blood count within 1 week before surgery; CA125 and HE4 levels within 2 weeks before surgery); (3) postoperative pathology (FIGO 2009 stage, histologic type). Four lymphocyte-based inflammatory indices were calculated as follows: NLR (neutrophil count/lymphocyte count), MLR (monocyte count/lymphocyte count), PLR (platelet count/lymphocyte count), and SII (neutrophil count × platelet count/lymphocyte count).

### Statistical methods

SPSS 29.0 and MedCalc 20.1.4 software were used to analyze the data. Measurement data were expressed as mean±standard deviation (x̄±s) for normally distributed data or median and interquartile range (IQR) for non-normal distributions. Intergroup comparisons were conducted using t-tests, Mann-Whitney U tests, or chi-square tests. Binary logistic regression was employed to develop a combined diagnostic model. Receiver operating characteristic (ROC) curve analysis was performed using MedCalc 20.1.4 software to evaluate diagnostic performance. The area under the curve (AUC) was calculated to assess the discriminatory power of individual indices and their combinations. Optimal cut-off values were determined by maximizing Youden's index, and corresponding sensitivity and specificity were computed for each model. Statistical significance was set at p < 0.05.

## Results

### Comparison of baseline data and markers between EOC and benign ovarian tumors

This study enrolled 391 patients with ovarian tumors, comprising 189 EOC cases and 202 benign controls. The EOC cohort included the following histologic subtypes: high-grade serous carcinoma (n = 141, 74.6%), clear cell carcinoma (n = 27, 14.3%), endometrioid carcinoma (n = 11, 5.8%), mucinous carcinoma (n = 7, 3.7%), carcinosarcoma (n = 2, 1.1%), and anaplastic carcinoma (n = 1, 0.5%). Benign tumors consisted of endometrioid tumors (n = 64, 31.7%), teratomas (n = 43, 21.3%), serous cystadenomas (n = 47, 23.3%), mucinous cystadenomas (n = 27, 13.4%), and other benign pathologies (n = 21, 10.4%). There were significant differences in age, body mass index (BMI), CA125, HE4, NLR, PLR, MLR, and SII between the two groups (P < 0.001). Serum concentrations of CA125, HE4, NLR, PLR, MLR, and SII were significantly higher in the EOC group than in the benign ovarian tumor group (P < 0.001) (Table 2).

### Correlation between CA125, HE4, NLR, PLR, MLR, SII, and clinicopathological features in patients with EOC

A comprehensive analysis of clinicopathological features, including age, BMI, menopausal status, FIGO staging, and pathological types, was performed in 189 patients with EOC. There was no statistically significant difference in CA125, HE4, NLR, PLR, MLR, and SII between different age groups (P > 0.05). No statistically significant differences were observed in CA125, HE4, NLR, MLR, or SII levels across different BMI groups (all P > 0.05). However, PLR values showed a significant difference between the BMI < 25 and BMI ≥ 25 groups (P = 0.015). There were no significant differences in tumor markers and inflammatory markers between menopausal and non-menopausal patients (P > 0.05). CA125 and HE4 were significantly different in early and advanced stages, and CA125 and HE4 were significantly higher in advanced stages than in early stages (P = 0.000, P = 0.012). NLR, PLR, MLR, and SII had no significant difference in early and advanced stages (P > 0.05). There were no significant differences in CA125, HE4, NLR, PLR, MLR, and SII among different pathological types (P > 0.05) (Table 3).

**Figure 1:**
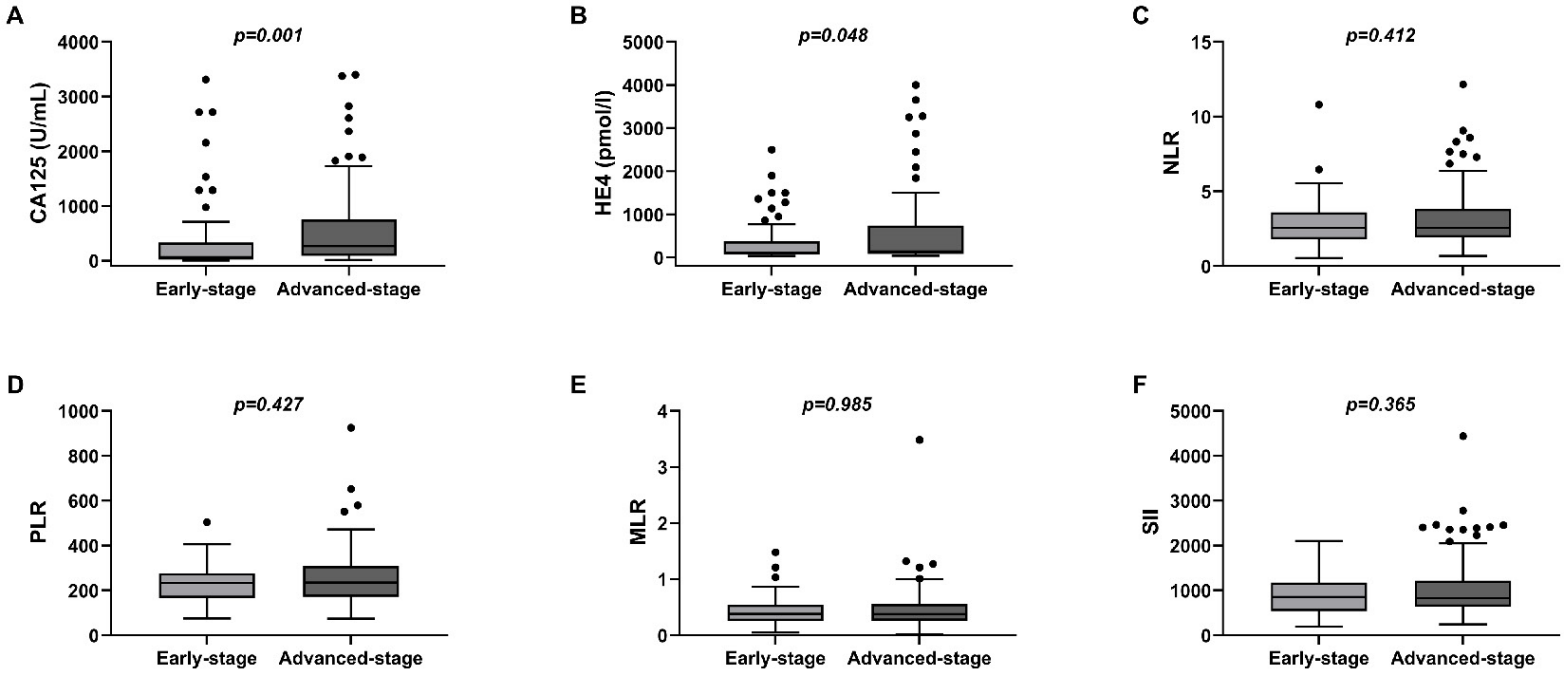
Box of the levels of peripheral blood CA125, HE4, NLR, PLR, MLR, and SII in early-stage EOC (Stage I-II) group and advanced-stage EOC (Stage III-IV) group. **(A)**CA125; **(B)**HE4; **(C)**NLR; **(D)**PLR; **(E)**MLR; **(F)**SII.

In 141 patients with high-grade serous ovarian cancer, CA125 and HE4 were significantly higher in the advanced stage than in the early stage (P = 0.001, P = 0.048), while there were no statistically significant differences in NLR, PLR, MLR, and SII between them (P > 0.05) (Figure 1).

### Efficacy of tumor and inflammatory markers in the diagnosis of EOC alone and in combination

The ROC curve of each index was drawn by binary logistic regression combined with inflammatory indicators and tumor markers. For assessing its diagnostic effectiveness, the AUC of the index was determined. Upon the Youden index hitting its peak, an optimal cut-off value was chosen. The MedCalc software was employed for determining the cut-off value, sensitivity, and specificity.

**Figure 2:**
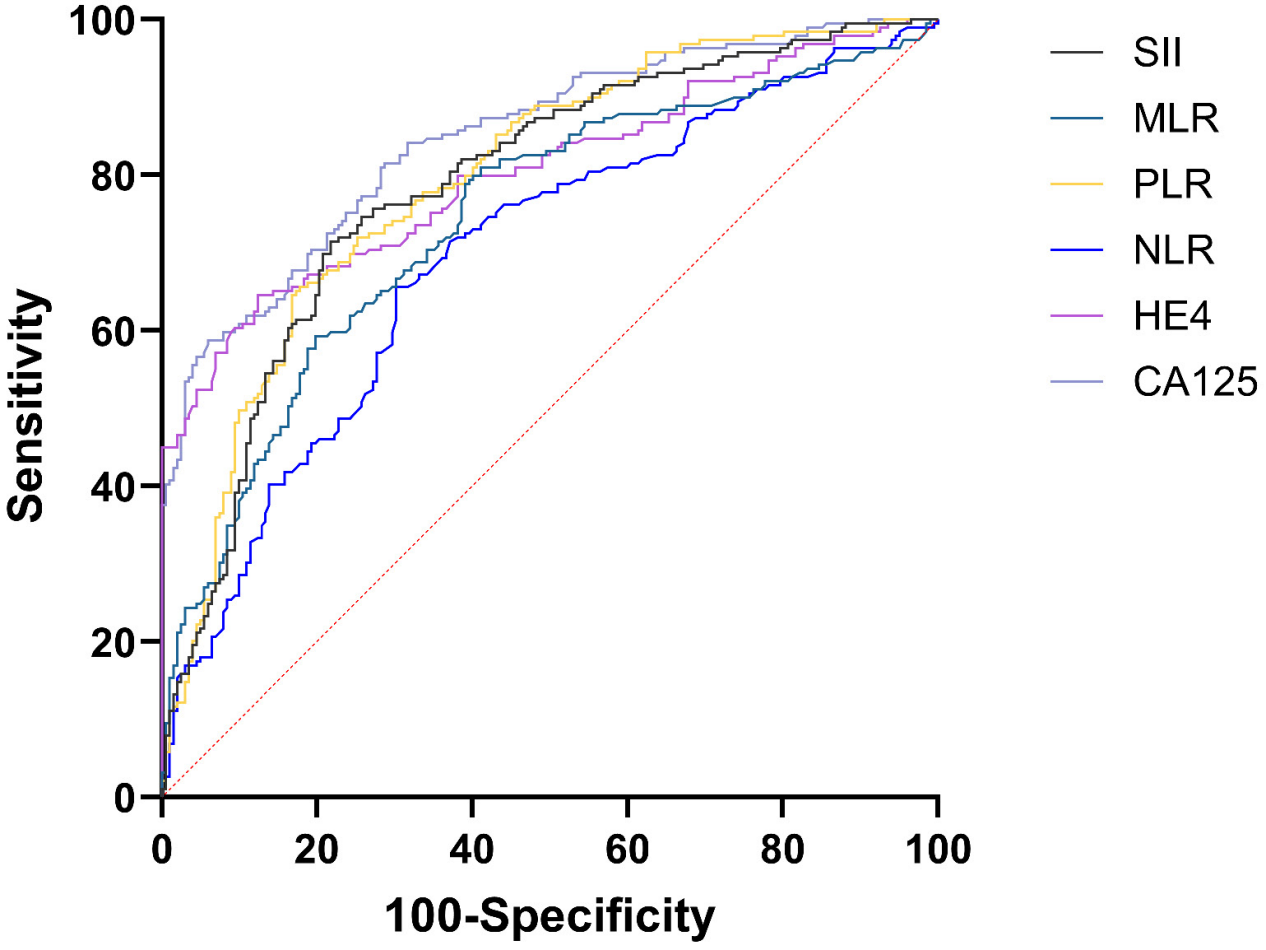
ROC curves of preoperative peripheral blood CA125, HE4, NLR, PLR, MLR, and SII for the diagnosis of EOC alone.

**Figure 3:**
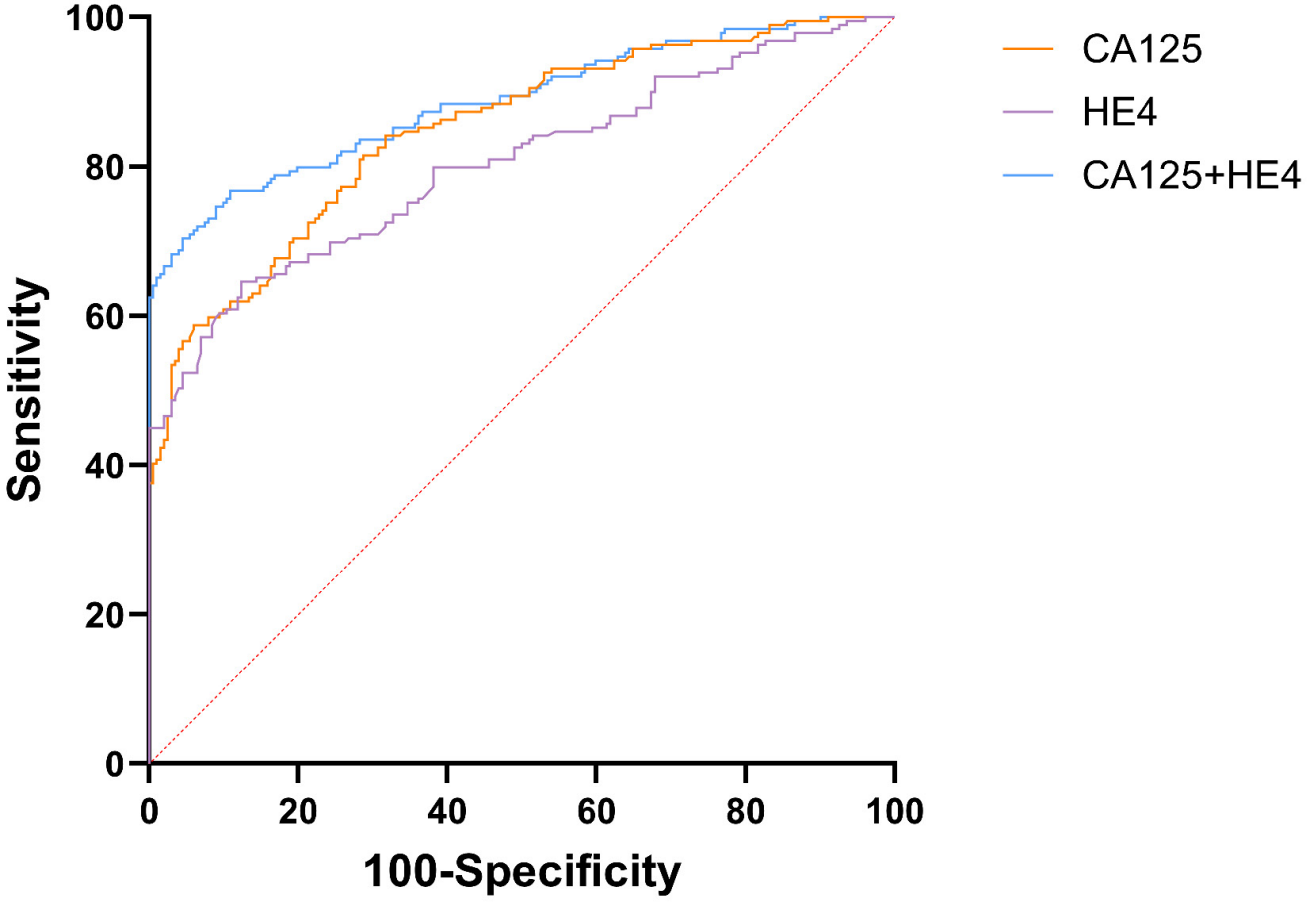
ROC curve of preoperative peripheral blood CA125 and HE4 combined for the diagnosis of EOC.

**Figure 4:**
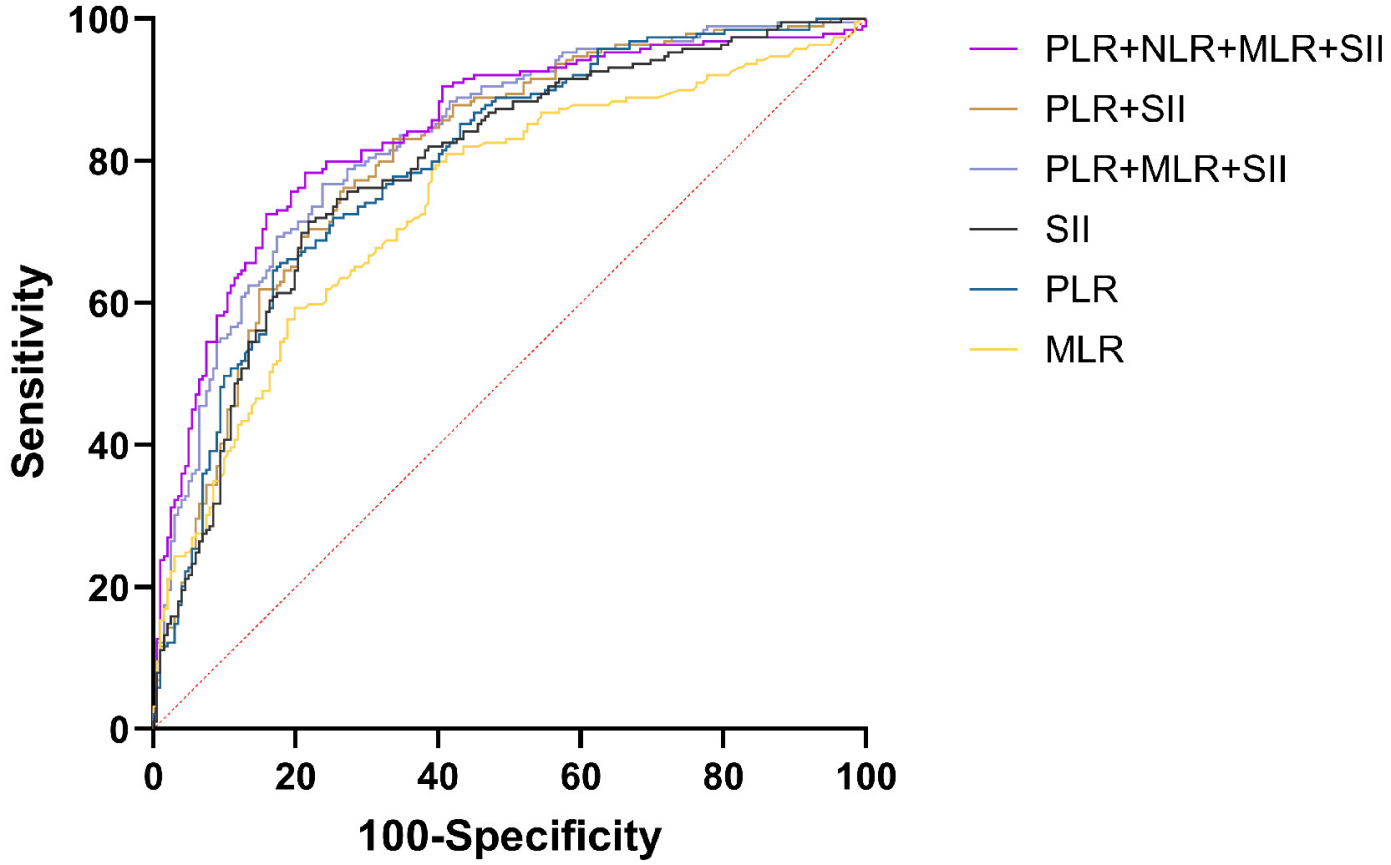
Efficacy analysis of NLR, PLR, MLR, and SII combined in the diagnosis of EOC.

The AUC for CA125 in diagnosing EOC stood at 0.848 (95%CI: 0.809-0.882), compared to HE4's 0.803 (95%CI: 0.760-0.841). Both CA125 and HE4 showed excellent predictive effects (Figure 2). The AUC for combined CA125+HE4 stood at 0.886 (95%CI: 0.850-0.916), with a cut-off value of 0.5388. Notably, the AUC for CA125+HE4 in EOC identification was substantially greater compared to individual CA125 and HE4 diagnoses (P = 0.0218, P < 0.0001) (Figure 3). The AUC for NLR+PLR+MLR+SII in diagnosing EOC stood at 0.840 (95%CI: 0.800-0.875), which was notably greater than those for NLR, PLR, MLR, and SII (P < 0.0001, P = 0.0037, P < 0.0001, P = 0.0048) (Figure 4).

**Figure 5:**
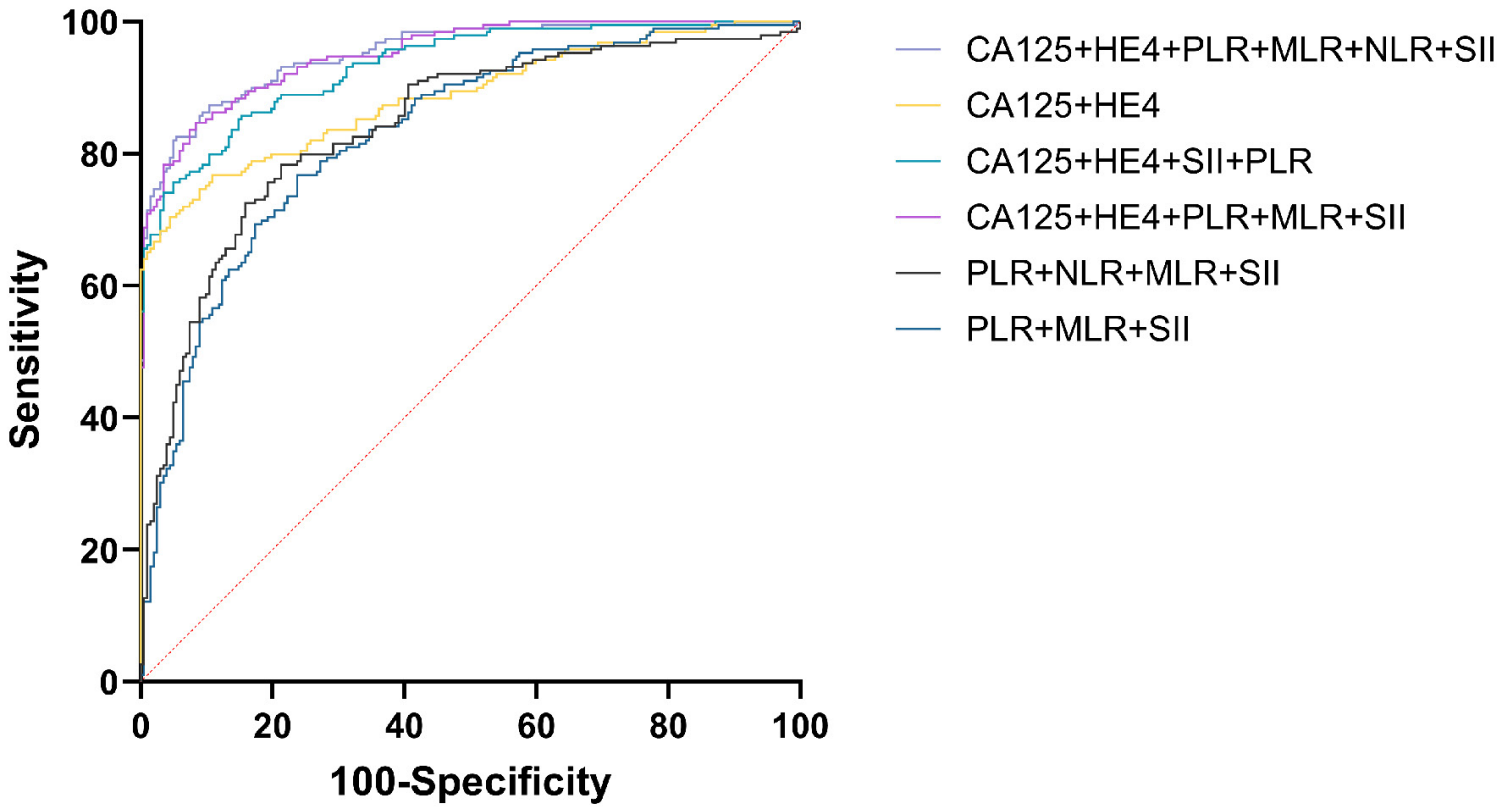
Efficacy analysis of CA125, HE4, NLR, PLR, MLR, and SII combined in the diagnosis of EOC.

For diagnosing EOC, CA125, HE4, either singly or alongside NLR, PLR, MLR, and SII were employed, achieving the greatest AUC for CA125+HE4+NLR+PLR+MLR+SII (AUC = 0.951) and CA125+HE4+PLR+MLR+SII (AUC = 0.951) respectively. NLR represents the lowest AUC at 0.696. The difference in AUC between CA125+HE4+NLR+PLR+MLR+SII and CA125+HE4+PLR+MLR+SII for diagnosing EOC was not statistically significant (P = 0.9305), but there was a statistically significant difference in AUC when compared with other tumor and inflammatory markers for diagnosing EOC (P < 0.05) (Figure 5).

To further compare the efficacy of the combination of tumor markers and inflammatory markers in the diagnosis of EOC, we evaluated the sensitivity and specificity of each indicator (Table 4). The highest sensitivity was CA125+HE4+PLR+MLR+SII (84.66%), and the highest specificity was CA125+HE4 (95.54%). Considering that NLR had the lowest AUC and limited diagnostic efficiency, CA125+HE4+PLR+MLR+SII was the optimal combination. The cut-off value of CA125+HE4+PLR+MLR+SII was 0.4107. Table 4 presents the AUC, cut-off value, sensitivity, and specificity of each index.

## Discussion

EOC, the most lethal gynecological malignancy, demonstrates poor early detection rates and dismal advanced-stage outcomes. In our cohort (n = 189), early-stage disease (FIGO I-II) accounted for 41.8% of cases, while advanced stages (III-IV) predominated (57.2%). The median age for EOC was 56 years, with postmenopausal patients outnumbering those without menopause.

EOC patients exhibited significantly higher BMI values compared to benign tumor controls (24.35±4.06 vs. 22.88±3.61kg/m², P < 0.001), consistent with existing epidemiological evidence linking obesity to carcinogenesis 12. There are numerous risk indicators for OC, with their biological mechanisms still not entirely comprehended. Although CA125 and HE4 are established biomarkers in OC clinical practice, their diagnostic performance remains suboptimal for early-stage disease due to limited sensitivity and moderate specificity in contemporary studies 13. Given the limitations of current biomarkers, the development of complementary diagnostic biomarkers is clinically imperative. Growing evidence underscores the pivotal role of chronic inflammation in oncogenesis, with systemic inflammatory indices emerging as promising adjuncts for early detection 14. Systemic inflammatory markers—quantifiable through routine blood tests—offer distinct advantages: minimal invasiveness, cost-effectiveness, and high reproducibility. This study innovatively integrates ovarian tumor markers (CA125/HE4) with systemic inflammatory indices (NLR, PLR, MLR, SII) to enhance early OC detection accuracy and ultimately improve patient outcomes.

Inflammation can promote tumor growth and development by releasing leukocytes and other phagocytic mediators or inflammatory cytokines to induce DNA damage, inhibit cell apoptosis, and promote angiogenesis in the surrounding area 15. In contrast to using only one blood indicator for inflammation, composite markers such as NLR, PLR, MLR, and SII demonstrate superior sensitivity and stability, simplifying the assessment of inflammation levels. Earlier research indicates the effectiveness of pre-surgical inflammation score assessments in diagnosing OC 16-18. Irina Balescu et al. 19 proposed that significant worsening of severe OC was linked to CA125 > 780µ/mL, NLR ≥ 2.7, MLR > 0.25, PLR > 200, and SII ≥ 84,1000. Multivariate analysis revealed that both MLR and SII were closely correlated with increased overall survival rates. Liyun Song et al. 20 suggested that CA125, HE4, SII, fibrinogen to albumin ratio (FAR), and MLR levels were significantly increased from ovarian borderline tumors to early OC. Prognostic nutritional index (PNI), NLR, PLR, MLR, SII, and FAR have excellent diagnostic performance for OC. However, current evidence on inflammation-based scores for ovarian cancer prediction is sparse, and the inflammatory markers evaluated in prior studies lack diversity and systematic validation. Data regarding the combination of tumor indicators and inflammatory markers is lacking, particularly in terms of the inflammatory score's diagnostic effectiveness in OC. Considering the diagnostic value of CA125 and HE4 and the hot spot status of systemic inflammatory markers, this study retrospectively enrolled 189 EOC and 202 benign ovarian tumors to explore the diagnostic value of tumor markers combined with inflammatory markers for EOC. The study aimed to determine the most effective combination, cut-off value, sensitivity, and specificity, thereby enhancing the evidence for better clinical practice. When assessing the levels of CA125, HE4, NLR, PLR, MLR, and SII in both the preoperative blood of EOC and benign ovarian tumor cohorts, it was observed that the EOC group exhibited significantly elevated measurements of CA125, HE4, NLR, PLR, MLR, and SII compared to the benign ovarian tumor group. Consistent with the majority of research findings 16, 21, the data indicates the significant importance of serum CA125, HE4, NLR, PLR, MLR, and SII in identifying EOC. Additionally, the ROC graph depicted the AUC for cancer and inflammation markers in EOC progression from high to low, marked as CA125 0.848, HE4 0.803, PLR 0.795, SII 0.788, MLR 0.742, and NLR 0.696 respectively. The optimal cut-off values were CA125 132 U/mL, HE4 99.78 U/mL, NLR 2.25, PLR 192.44, MLR 0.225, and SII 632.84. Tumor markers (CA125: AUC = 0.848; HE4: AUC = 0.803) significantly outperformed inflammatory ratios (PLR: 0.795; SII: 0.788; MLR: 0.742; all p < 0.05), establishing their primacy in EOC diagnosis. Dochez V et al. 22 found that the average values of HE4 and CA125 in the OC group were significantly higher than those in the benign group. In relation to using either HE4 or CA125 solo, the specificity of HE4 and CA125 together was significantly higher (99.5%), mirroring that observed in this own investigation. The NLR factor has consistently been identified as significant in predicting outcomes in advanced OC or other malignant tumors 23, 24. Yoshida A et al. 25 considered that in the identification of ovarian masses, the correlation of CA125 or NLR was 71.09% and 73.89%, achieving the optimal balance in sensitivity and specificity. However, in this study, compared with MLR, PLR, SII, and other inflammatory markers, the AUC of NLR was 0.696, and the diagnostic efficacy was poor.

During tumor formation, lymphocytes migrate into the tumor microenvironment, differentiate into tumor-infiltrating lymphocytes, and mediate anti-tumor immunity by inducing cytotoxic cell death, thereby inhibiting tumor cell proliferation and migration 26-28. A reduced lymphocyte count is associated with unfavorable oncologic outcomes. Given their proven role in tumor defense, lymphocytes serve as biomarkers for OC diagnosis and prognosis, with their values contributing to NLR, MLR, and SII calculations 29, 30. Platelets may contribute to anti-tumor immunity through inflammatory responses. However, preoperative thrombocytosis-frequently observed in high-grade serous OC 31, 32-suggests a complex role in cancer progression. Pro-inflammatory cytokines (e.g., IL-1, IL-6) drive thrombocytosis 33, 34, and elevated platelet counts coupled with altered PLR values may reflect both systemic inflammation and tumor microenvironment modulation 35. Monocytes can be recruited to tumor tissues and differentiate into tumor-associated macrophages (TAMs). TAMs then release epidermal growth factors and angiogenic factors to promote tumor cell proliferation and migration 36. Tang Y et al. demonstrated that the combined use of LMR and CA125 before surgery can predict OC outcomes 37, and they also established an association between LMR, used either solely or alongside serum CA125, and the stage of OC progression 38. Fulvio Borella et al. 39 conducted a retrospective, single-center, observational study to evaluate inflammatory markers by analyzing blood samples collected at initial diagnosis before EOC surgery. In univariate analysis, NLR, PLR, and SII were all associated with disease-free survival and cancer-specific survival. In multivariate Cox analysis, SII was an independent predictor of disease-free survival. Previous studies have indicated potential associations between systemic inflammatory markers (NLR, MLR, PLR, SII) and other markers with OC prognosis. However, standardized cut-off values are lacking, individual markers exhibit limited diagnostic power, and robust models combining inflammatory and tumor markers remain scarce. Large-scale, multi-center studies are required for further validation. Our study further analyzed ROC curves for the combined use of multiple indicators. For diagnosing EOC, both CA125+HE4+NLR+PLR+MLR+SII (AUC = 0.951) and CA125+HE4+PLR+MLR+SII (AUC = 0.951) achieved the greatest AUC. The difference in AUC between CA125+HE4+NLR+PLR+MLR+SII and CA125+HE4+PLR+MLR+SII for diagnosing EOC was not statistically significant (P = 0.9305). The combination of CA125+HE4+PLR+MLR+SII demonstrated the highest sensitivity (84.66%), while CA125+HE4 showed the highest specificity (95.54%). Given that CA125+HE4+PLR+MLR+SII achieved the greatest AUC with a simplified panel of biomarkers and no statistically significant improvement from adding NLR (P = 0.9305), this combination represents the optimal diagnostic model, offering the highest efficacy and sensitivity for improved clinical detection of EOC.

This study has several limitations. First, as a retrospective analysis involving patients with EOC and benign ovarian diseases, it may not fully account for potential confounding factors that could introduce bias. Second, the single-center design and relatively small sample size may limit the generalizability of our findings to broader populations. Therefore, future multi-center studies with larger cohorts are warranted to validate the diagnostic value of combining tumor markers (CA125 and HE4) with inflammatory indicators (PLR, MLR, and SII) for EOC detection. Nevertheless, our results demonstrate that the CA125+HE4+PLR+MLR+SII panel exhibits superior diagnostic performance, showing the highest efficiency and sensitivity with excellent diagnostic accuracy for EOC. These findings provide valuable evidence to support its potential clinical application in early EOC diagnosis.

## Conclusion

In conclusion, our study demonstrates that the combined use of preoperative peripheral blood tumor markers and inflammatory biomarkers significantly enhances the diagnostic accuracy of EOC. Notably, the CA125+HE4+PLR+MLR+SII panel exhibited optimal diagnostic performance, achieving the highest sensitivity and diagnostic efficacy among all evaluated combinations. These findings provide a clinically applicable approach for early EOC detection and establish a reliable foundation for diagnostic decision-making. This integrated diagnostic strategy warrants further validation through multicenter prospective studies.

## Supplementary Material

Supplementary data.

## Figures and Tables

**Table 1:** Definitions and main functions of NLR, MLR, PLR and SII in cancer studies.

Index	Definition	Main functions
NLR	neutrophil count/lymphocyte count	Neutrophils drive tumor progression via inflammation, angiogenesis, and metastasis, while also suppressing antitumor immunity. Lymphocytes are critical for tumor cell killing, though immunosuppressive subsets may counteract this effect. The NLR integrates these roles, serving as a biomarker for tumor immune status and patient prognosis.
MLR	monocyte count/lymphocyte count	Monocyte differentiation into tumor-associated macrophages promotes tumor immune escape and metastasis. MLR serves as a composite indicator of immunosuppression and impaired antitumor immunity, which can predict tumor prognosis.
PLR	platelet count/lymphocyte count	PLR reflects platelet-driven tumor metastasis, angiogenesis, and immunosuppression, and correlates with poor clinical outcomes, therapy resistance, and advanced disease progression.
SII	neutrophil count ×platelet count/lymphocyte count	SII quantifies the balance between pro-inflammatory drivers and immunosuppression, reflecting the systemic inflammatory state.

**Table 2:** Comparison of defined variables between EOC and benign ovarian tumors.

Variables	N (%)	Age (yr), median (IQR)	BMI (kg/m^2^), (x̄±s)	CA125 (U/mL), median (IQR)	HE4 (pmol/L), median (IQR)	NLR, median (IQR)	PLR, median (IQR)	MLR, median (IQR)	SII, median (IQR)
EOC	189 (48.34%)	56(18)	24.35±4.06	174.40 (531.75)	124.90 (470.62)	2.58 (1.80)	232.52 (122.47)	0.38 (0.29)	848.23 (606.77)
Benign ovarian tumors	202 (51.66%)	38(15)	22.88±3.61	16.97 (26.96)	62.23 (37.02)	1.85 (1.19)	129.97 (77.09)	0.19 (0.19)	439.08 (292.49)
Z-value		-10.019		-11.906	-10.345	-6.698	-10.101	-8.288	-9.836
t-value			3.781						
P-value		<0.001	<0.001	<0.001	<0.001	<0.001	<0.001	<0.001	<0.001

EOC, epithelial ovarian cancer; BMI, body mass index; NLR, neutrophil count/lymphocyte count; MLR, monocyte count/lymphocyte count; PLR, platelet count/lymphocyte count; SII, neutrophil count × platelet count/lymphocyte count; IQR, interquartile range.

**Table 3:** Relationship between tumor and inflammatory markers and clinicopathological features in patients with EOC.

Variables	N (%)	CA125 (U/mL), median (IQR)	HE4 (pmol/L), median (IQR)	NLR, median (IQR)	PLR, median (IQR)	MLR, median (IQR)	SII, median (IQR)
Age							
≤ 50	67 (35.45%)	179.40 (489.67)	170.01 (466.34)	2.54 (1.56)	246.10 (121.55)	0.43 (0.25)	881.58 (607.76)
> 50	122 (64.55%)	169.97 (597.97)	116.50 (464.13)	2.59 (1.98)	215.75 (126.49)	0.36 (0.29)	825.33 (633.90)
Z-value		-0.083	-1.123	-0.249	-1.450	-1.814	-0.820
P-value		0.934	0.262	0.805	0.148	0.070	0.414
BMI							
< 25	110 (58.20%)	165.17 (441.95)	129.35 (463.30)	2.58 (2.00)	203.21 (118.71)	0.36 (0.28)	837.67 (636.57)
≥ 25	79 (41.80%)	194.50 (535.61)	122.70 (614.18)	2.59 (1.51)	246.10 (111.53)	0.41 (0.34)	867.94 (577.11)
Z-value		-1.294	-0.348	-0.003	-2.434	-1.197	-0.944
P-value		0.196	0.729	0.998	0.015	0.232	0.347
Menopausal state							
Yes	114 (60.32%)	178.40 (657.09)	116.75 (411.66)	2.76 (2.02)	223.27 (124.88)	0.375 (0.31)	861.37 (588.53)
No	75 (39.68%)	161.03 (429.26)	168.70 (472.89)	2.47 (1.58)	234.97 (107.77)	0.392 (0.28)	818.64 (633.96)
Z-value		-1.027	-0.485	-0.773	-0.018	-0.397	-0.117
P-value		0.305	0.629	0.441	0.986	0.693	0.908
FIGO staging							
I-II	79 (41.80%)	64.39 (305.78)	111.70 (312.03)	2.56 (1.80)	232.52 (109.72)	0.38 (0.28)	854.79 (635.78)
III-IV	110 (58.20%)	267.05 (701.25)	145.15 (668.54)	2.58 (1.83)	229.37 (140.30)	0.38 (0.30)	837.67 (550.34)
Z-value		-4.49	-2.50	-1.04	-0.70	-0.19	-0.93
P-value		0.000	0.012	0.300	0.486	0.849	0.354
Pathological type							
Serous	141 (74.60%)	179.40 (548.60)	134.90 (470.78)	2.58 (1.65)	234.21 (123.23)	0.378 (0.30)	857.36 (606.77)
Clearcell	27 (14.29%)	177.40 (544.07)	116.40 (484.23)	2.73 (3.65)	241.67 (137.18)	0.392 (0.30)	896.37 (899.49)
Endometrioid	11 (5.82%)	194.50 (914.23)	121.50 (368.40)	2.58 (1.68)	210.14 (65.31)	0.383 (0.30)	787.45 (435.99)
Others	10 (5.29%)	45.53 (51.43)	87.22 (205.99)	1.96 (1.46)	225.09 (82.07)	0.413 (0.22)	633.90 (560.74)
H (K)		5.443	3.698	0.480	3.882	1.015	2.274
P-value		0.142	0.296	0.923	0.274	0.798	0.518

BMI, body mass index; NLR, neutrophil count/lymphocyte count; MLR, monocyte count/lymphocyte count; PLR, platelet count/lymphocyte count; SII, neutrophil count × platelet count/lymphocyte count; IQR, interquartile range; Others, mucinous carcinoma, carcinosarcoma, and anaplastic carcinoma.

**Table 4:** Efficacy of CA125, HE4, NLR, PLR, MLR, SII, and the combination of them in the diagnosis of EOC.

Indicators	AUC	95%CI	Cut-off	Youden index	Sensitivity (%)	Specificity (%)
CA125	0.848	0.809~0.882	132 U/mL	0.5279	58.73	94.06
HE4	0.803	0.760~0.841	99.78 U/mL	0.5217	64.55	87.62
NLR	0.696	0.648~0.741	2.25	0.3541	65.61	69.80
PLR	0.795	0.752~0.834	192.44	0.4779	65.61	82.18
MLR	0.742	0.696~0.785	0.225	0.3986	80.95	58.91
SII	0.788	0.744~0.827	632.84	0.4965	71.43	78.22
CA125+HE4	0.886	0.850~0.916	0.5388	0.6591	70.37	95.54
NLR+PLR+MLR+SII	0.840	0.800~0.875	0.4399	0.5702	78.31	78.71
PLR+MLR+SII	0.830	0.789~0.866	0.4447	0.5296	76.72	76.24
PLR+SII	0.806	0.763~0.844	0.4300	0.4946	76.19	73.27
CA125+HE4+NLR+PLR+MLR+SII	0.951	0.925~0.970	0.5186	0.7709	82.54	94.55
CA125+HE4+PLR+MLR+SII	0.951	0.924~0.970	0.4107	0.7624	84.66	91.58
CA125+HE4+ PLR+SII	0.933	0.904~0.956	0.5797	0.7071	75.66	95.05

NLR, neutrophil count/lymphocyte count; MLR, monocyte count/lymphocyte count; PLR, platelet count/lymphocyte count; SII, neutrophil count × platelet count/lymphocyte count; AUC, the area under the curve.

## Abbreviations

CA125: cancer antigen 125
HE4: human epitope protein 4
NLR: neutrophil-to-lymphocyte ratio
MLR: monocyte-to-lymphocyte ratio
PLR: platelet count-to-lymphocyte ratio
SII: systemic immunoinflammatory index
OC: ovarian cancer
EOC: epithelial ovarian cancer
FIGO: International Federation of Gynecology and Obstetrics
ROC: the receiver operating characteristic curve
AUC: the area under the curve
BMI: body mass index
